# Life-Saving Multidisciplinary Management of Self-Inflicted Tracheal Transection: A Case Report

**DOI:** 10.7759/cureus.65174

**Published:** 2024-07-23

**Authors:** Pooja Thaware, Saurabh Trivedi, Anshu Lakra, Ankita, Mohd Yunus

**Affiliations:** 1 Trauma and Emergency Medicine, All India Institute of Medical Sciences, Bhopal, Bhopal, IND

**Keywords:** trachea, self-injurious behavior, patient care team, depression, bronchoscopes

## Abstract

Self-inflicted cut-throat injuries, often associated with severe psychiatric disorders and exacerbated by socioeconomic factors, present significant medical complexities. Here, we present the case of a 45-year-old male with major depressive disorder who attempted suicide with a sword, resulting in complete tracheal transection. Upon admission, he presented with severe respiratory distress and hemorrhage, necessitating immediate fluid resuscitation and immediate airway securing with pediatric fiberoptic bronchoscopy, which successfully stabilized his airway. The surgical intervention included end-to-end tracheal anastomosis and T-tube placement without immediate complications. Postoperatively, the patient required intensive care with ventilator support and psychiatric intervention. Successful management of severe self-inflicted neck injuries relies on prompt airway control, precise surgical techniques, and comprehensive postoperative care to mitigate complications such as sepsis and tracheal stenosis.

## Introduction

Self-inflicted cut-throat injuries, though rare, present as critical medical emergencies with unique challenges in airway and surgical management. These incidents frequently occur in the context of severe psychiatric disorders and carry a high risk of morbidity and mortality. Clinical manifestations typically include significant bleeding, airway compromise, and hypovolemic shock. Effective management necessitates coordinated efforts among emergency physicians, anesthesiologists, ENT specialists, and psychiatrists to ensure successful airway control, surgical repair, prevention of postoperative complications, such as sepsis, and ongoing psychiatric care to address underlying mental health issues [1]. We present a case report of a 45-year-old male suffering from depression and non-compliance with medication who sustained a self-inflicted cut-throat injury resulting in the complete transection of the trachea. Despite significant challenges in securing the airway, prompt management was initiated in the emergency department. The patient was immediately transferred to the operating theater for surgical intervention, followed by intensive care unit management for one month. Intensive care and mental health counseling were provided, leading to the patient's discharge in improved clinical and mental health status.

## Case presentation

**Figure 1:**
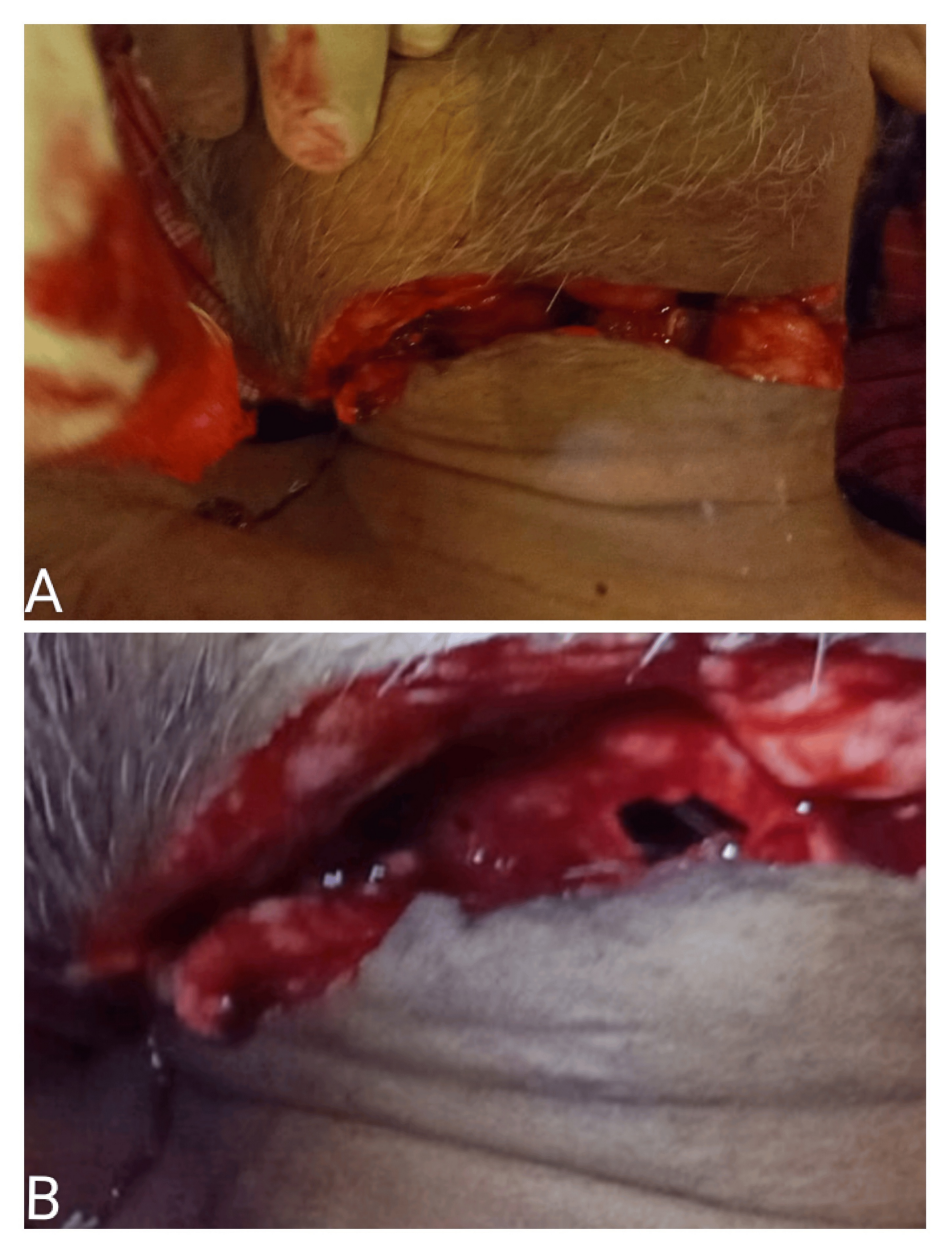
(A) Self-inflicted cut-throat injury. (B) Complete transection of trachea due to cut-throat injury.

A 45-year-old male clerk, diagnosed with major depressive disorder following the tragic loss of his 15-year-old child in a road accident a year prior, presented with a self-inflicted neck injury using a sword (Figure 1). The patient was non-compliant with medication and had irregular follow-ups at the psychiatric outpatient department of another hospital, according to the history provided by the relatives. Additionally, no documentation was available. Over the preceding two months, he had ceased working due to worsening symptoms. Discovered by family members in his bedroom, he was promptly transported to the emergency department. On arrival, the patient exhibited respiratory distress with an open neck wound and a complete transection of the trachea just above the vocal cords. Despite the tracheal injury, there was no damage to the neck vessels.

The injury precluded conventional oral intubation due to the tracheal transection. The patient was hypotensive due to continuous bleeding from the wound; initial stabilization included securing two large-bore IV cannulas and administering 1 L of Ringer's lactate solution. This stabilized the mean arterial pressure, with blood typing and cross-matching preparations made for emergent surgical intervention.

To secure the airway, 10% lignocaine was applied to the vocal cords. Despite challenges, a bougie was cautiously advanced through the open wound and vocal cords; it went in with difficulty. The patient was sedated with ketamine (1 mg/kg), followed by unsuccessful attempts to pass a 7-mm endotracheal tube over the bougie. A smaller 6.5-mm endotracheal tube also failed to negotiate the airway. Oxygenation was maintained via an oxygen tube over the transected trachea and Hudson’s mask, sustaining oxygen saturation above 94%.

A pediatric fiberoptic bronchoscope with a 6-mm endotracheal tube was swiftly utilized, revealing a sizable blood clot approximately 3-4 cm below the vocal cords obstructing tube insertion. The clot was extracted using pediatric Magill forceps under direct bronchoscopic guidance. Subsequently, a 6.5-mm endotracheal tube was successfully passed through the transected wound, securing the airway. The patient was then paralyzed with 6-mg IV vecuronium and initiated on fentanyl infusion for sedation before being expedited to the operating room.

**Figure 2:**
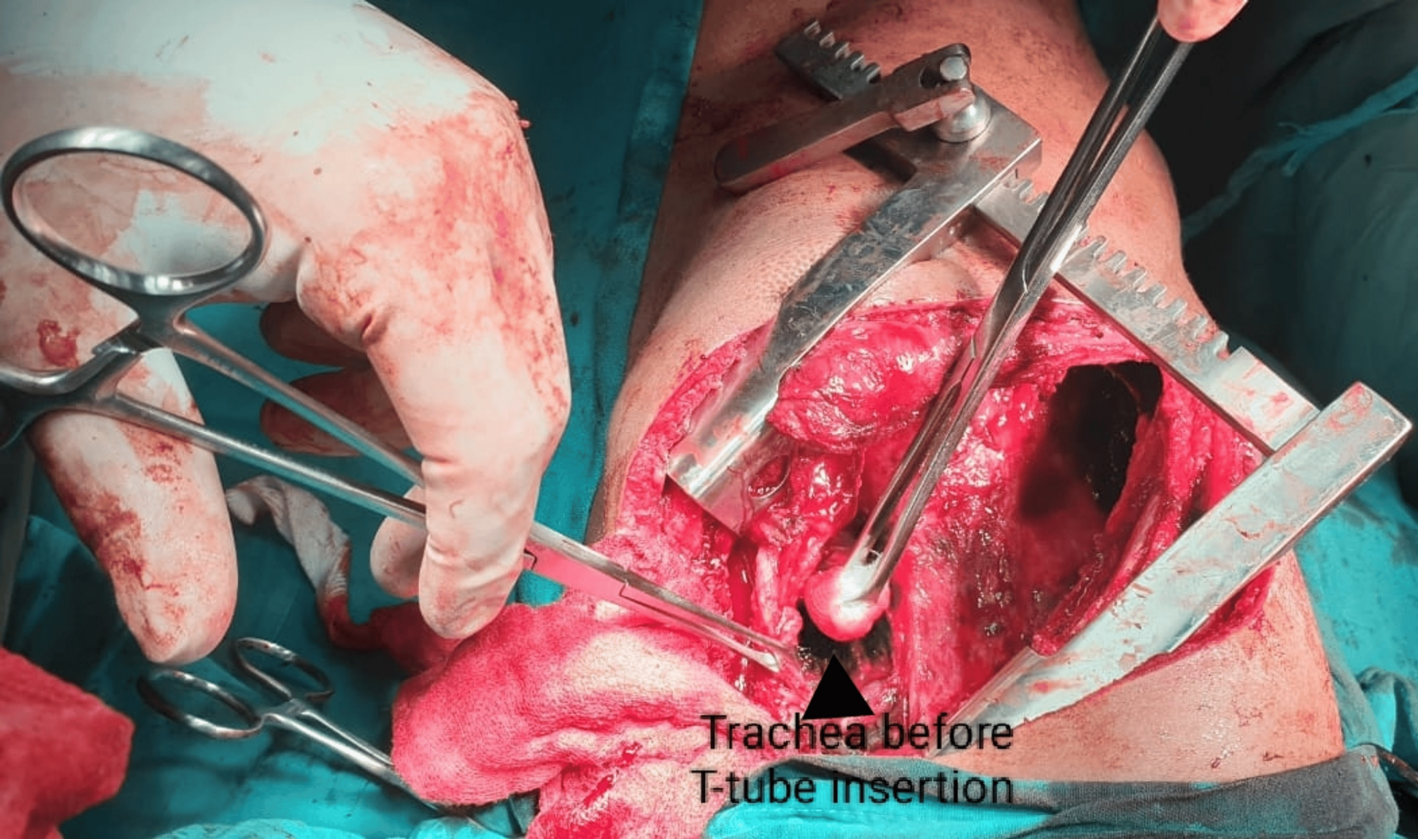
Surgical neck exploration showing trachea before placement of T-tube.

In the operating theater, ENT specialists performed neck exploration, confirming complete tracheal transection with intact neck vasculature (Figure 2). An end-to-end tracheal anastomosis and T-tube placement were executed without immediate complications. Postoperatively, the patient was closely monitored in the ICU, with psychiatric medications continued and ventilator weaning achieved by postoperative day 3. The first psychiatric evaluation was conducted on postoperative day 4, and the patient's ongoing medication (divalproex and olanzapine) was optimized. Continued weekly evaluations revealed that the patient had an exacerbation of his psychotic (persecutory) symptoms, attributed to poor compliance. A final psychiatric diagnosis of Psychosis Not Otherwise Specified (NOS) was made, and it was decided to continue monitoring the patient's psychotic symptoms post-discharge. With continued intensive critical care and psychiatric counseling, we were able to discharge the patient with a T-tube on postoperative day 30.

## Discussion

Self-inflicted cut-throat injuries are primarily associated with psychiatric disorders such as major depressive disorder, schizophrenia, and personality disorders, often compounded by intoxication [2-4]. Socioeconomic factors, such as unemployment, social isolation, and financial stress, also significantly contribute to these incidents. A retrospective study conducted at Chattogram Medical College Morgue House from 2014 to 2016 revealed that most victims were males aged 21-30 years, with hemorrhage being the leading cause of death. Butcher's knives were the most commonly used weapon [5].

Patients with self-inflicted cut-throat injuries typically present with severe neck wounds involving the trachea, esophagus, blood vessels, and nerves, as evidenced by various case reports demonstrating the variable extent of injury [6]. The primary challenge in managing these injuries lies in securing the airway, as traditional oral intubation may prove difficult or impossible due to the nature of the injury. Alternative techniques, such as inserting an endotracheal tube through the open tracheal wound or using a bougie for guidance, have been successfully employed in several cases [7].

This case underscores the urgency and complexity involved in managing severe self-inflicted neck injuries. The horizontal orientation of the wound in this instance was atypical, as self-inflicted injuries typically manifest with oblique wounds. The complete tracheal transection necessitated immediate and adept airway management techniques, highlighting the critical role of flexible emergency protocols.

Upon immediate assessment of the tracheal transection, arrangements were swiftly made for tools including a bougie, smaller diameter endotracheal tubes, and adult and pediatric fiberoptic bronchoscopes. The decision to use a pediatric fiberoptic bronchoscope proved pivotal, saving valuable time and improving patient outcomes. The management of airway challenges is paramount, with quick intervention pivotal in saving lives.

Surgical management typically involves meticulous exploration of the neck wound, hemorrhage control, and repair of damaged structures. Procedures, such as end-to-end tracheal anastomosis and T-tube placement, are common and aimed at restoring tracheal continuity, maintaining airway patency, and preventing infection.

Postoperative care is critical due to the high risks of complications, such as sepsis, wound infections, and tracheal stenosis. Intensive ICU monitoring and management are often necessary. Despite successful initial management, the ongoing risk of complications remains significant. Fortunately, our patient survived and was discharged in improved condition with continued medication. Recognizing the family's limited support due to educational gaps, they were counseled during hospitalization on providing adequate mental support and ensuring regular follow-up care.

The patient expressed profound gratitude to the medical team for providing a second chance at life.

## Conclusions

Patients with psychiatric illnesses often suffer in silence, particularly when there is a lack of education within the family regarding mental health. Such cases may present to emergency departments with self-inflicted life-threatening injuries. These patients require another chance at life, which was made possible in our case through immediate and prompt stabilization of the difficult airway in the emergency setting, followed by timely surgical intervention that reduced the risk of sepsis. Intensive critical care and dedicated efforts toward mental well-being were crucial in the patient’s recovery. The family was also educated to ensure continued care for the patient’s mental health. Multidisciplinary prompt management and holistic postoperative care are imperative in the successful treatment of self-inflicted injuries in psychiatric patients.
